# Usefulness of CogEvo, a computerized cognitive assessment and training tool, for distinguishing patients with mild Alzheimer's disease and mild cognitive impairment from cognitively normal older people

**DOI:** 10.1111/ggi.14110

**Published:** 2020-12-17

**Authors:** Hajime Takechi, Hiroshi Yoshino

**Affiliations:** ^1^ Department of Geriatrics and Cognitive Disorders Fujita Health University School of Medicine Toyoake Japan

**Keywords:** Alzheimer’s disease, cognitive training, computerized cognitive assessment, dementia, mild cognitive impairment

## Abstract

**Aim:**

This study aimed to assess whether CogEvo, a computerized cognitive assessment and training tool, could distinguish patients with mild Alzheimer's disease and mild cognitive impairment from cognitively normal older people.

**Methods:**

This cross‐sectional study enrolled 166 participants with Alzheimer's disease, mild cognitive impairment and cognitively normal older people. In CogEvo, five types of cognitive tasks were carried out, and the z‐scores were used as a composite score. Logistic regression and receiver operating characteristics analyses were then carried out to evaluate the usefulness of CogEvo in distinguishing between the three groups.

**Results:**

CogEvo and Mini‐Mental State Examination scores showed excellent correlation, and could significantly differentiate between the Alzheimer's disease, mild cognitive impairment and cognitively normal older people groups (Mini‐Mental State Examination 20.4 ± 3.5, 25.5 ± 1.6 and 27.6 ± 2.0, respectively; CogEvo: −1.9 ± 0.9, −0.8 ± 0.8 and 0.0 ± 1.0, respectively; both *P* < 0.001 by analysis of variance). Logistic regression analysis adjusted for age, sex and years of education significantly differentiated the mild cognitive dysfunction group (mild cognitive impairment plus mild Alzheimer's disease; *n* = 78) from the cognitively normal group (*n* = 88) (*P* < 0.001), whereas receiver operating characteristics analysis showed moderate accuracy (area under the receiver operating characteristic curve 0.830).

**Conclusions:**

These results suggest that CogEvo, a computerized cognitive assessment tool, is useful for evaluating early‐stage cognitive impairment. Further studies are required to assess its effectiveness as a combination assessment and training tool. **Geriatr Gerontol Int 2021; 21: 192–196**.

## Introduction

The prevalence of dementia is increasing with the aging of the population, and it is estimated that the number of patients with dementia worldwide will reach 46.8 million by 2030.^1^ As dementia disturbs the autonomy of patients in daily life and often requires nursing care, it is a major issue in terms of quality of life for these patients and their families.^2^ Dementia is also an important issue in terms of medical and social economics.^3^, ^4^

For the diagnosis of early dementia and mild cognitive impairment (MCI), it is important to carry out cognitive examinations; these are usually carried out in specialized medical institutions. However, the numbers of institutions and specialized persons who can carry out such examinations are limited. As the number of patients with dementia is expected to rise, the need for cognitive examinations is also expected to increase.^5^ Therefore, new methods for assessing cognitive status need to be developed.

In recent years, computer software has been increasingly used to assess cognitive function.^6^, ^7^, ^8^, ^9^, ^10^ Using a computer enables the test presentations to be indicated automatically, eliminating the need for a specialized tester; this could pave the way for Internet‐based cognitive examinations. In some computer‐based cognitive assessments, test presentations are indicated in different patterns for each implementation, so that the user can undergo the cognitive evaluations repeatedly without becoming fatigued.^11^ Taking advantage of this, cognitive function can be easily and continuously measured, similar to daily blood pressure measurements. Recently, computer‐based cognitive training systems have also been developed and tested to help maintain cognitive function.^12^, ^13^, ^14^, ^15^ However, to our knowledge, no computer software that can be used to screen for dementia in the early stage, as well as for daily cognitive training, has been developed. In the present study, we aimed to verify whether CogEvo, a software program developed for combined use as a cognitive function testing and training tool, is effective in screening for early‐stage dementia as a step to use it as a combination tool.

## Methods

### *Participants*

We recruited patients who had visited an outpatient memory clinic and been diagnosed in standard examinations as being cognitively normal (CN), having MCI or having mild Alzheimer's disease (AD). Those who accompanied the patients, usually a spouse, who were also judged as being CN were also recruited. Patients were diagnosed after undergoing a typical physical examination, cognitive assessment, blood test, head computed tomography or magnetic resonance imaging. For the cognitive assessment, the Mini‐Mental State Examination (MMSE), Scenery Picture Memory Test, Logical Memory Test of the Wechsler Memory Scale‐Revised, Clock Drawing Test, Word Fluency Test (vegetable, animal, letter “ka”), Trail Making Test and Block Design Test from the Wechsler Adult Intelligence Scale‐Revised were used.^16^ The Clinical Dementia Rating scale was also used for evaluations.^17^ The family member accompanying the patient was confirmed to be normal if they achieved a Clinical Dementia Rating score of 0, assessed by a certified nurse for dementia through intake regarding daily living of patients and care coordination by the family members. In addition, the cognitive status of family members was confirmed using the Scenery Picture Memory Test, which has high sensitivity and specificity for detecting early‐phase cognitive decline.^16^, ^18^ The diagnosis of AD was based on the National Institute of Aging and Alzheimer's Association probable AD criteria.^19^ For patients with MCI, only MCI due to AD was included. For the diagnosis of MCI due to AD, the National Institute of Aging and Alzheimer's Association MCI clinical diagnosis criteria were used.^20^ Patients diagnosed with cerebrovascular or Lewy body dementia, or other neurological or psychiatric disorders were excluded. This study was approved by the ethics committee of Fujita Health University (HM17‐244), and written, informed consent was obtained from all study participants.

### *CogEvo, a computerized cognitive assessment and training tool*

CogEvo (Total Brain Care, Kobe, Japan) is composed of 12 different types of tests, and the reliability and validity of the short version of CogEvo have been verified for older people.^11^, ^21^ For the purposes of the present study, CogEvo including five types of tests was used (“Orientation”, “Visual search”, “Flash light”, “Route 99” and “Just fit”).^21^ To select the five tasks, we held a consensus meeting of dementia specialists consisting of two psychiatrists, two neurologists and a geriatrician regarding the use of CogEvo as an early‐stage screening tool for dementia, where we discussed difficulties for older people, the meaning of cognitive assessment and the time of execution. After this meeting, the five tasks were considered appropriate and selected for the clinical trial. Very recently, CogEvo was introduced and shown to be useful to evaluate age‐related cognitive decline.^21^ Here, we briefly explain the five CogEvo test types used in the present study. In “Orientation”, participants are asked to select the correct date and time from among 14 choices on cards presented on a liquid‐crystal display monitor. This test asks about not only the present day, but also, for example, the day before yesterday. In “Visual search” or the “modified Trail Making Test”, participants select letters or numbers in alphabetical or numerical order as quickly as possible. For example, participants select letters or numbers in turn, such as “A, 1, B, 2, C, 3”. In “Flash light”, participants must click a button in order of the color of four flashing lights on the liquid‐crystal display monitor. After the colors stop glowing, the participants click the buttons in the correct color order. This task increases in difficulty until failure; for example, blue, yellow as the first step, and yellow, blue, red as the next step. In “Route 99”, participants must select all numbers shown in turn in 8 × 8 blocks in order. In “Just fit”, participants select the same figure as that shown in the center from six surrounding similar figure selections, with increasing complexity of the figures or the similarity between the target and choices. In these tests, “Orientation” is designed to evaluate time orientation, “Visual search” to evaluate executive function, “Flash light” to evaluate attention, “Route 99” to evaluate planning ability and “Just fit” to evaluate visuospatial recognition. A computer with a touch screen and stylus was used for test input. The examination time was approximately 10 min per person. CogEvo is equipped with audio and visual test instructions, and can be carried out by participants independently. However, in the present study, a trained instructor gave simple side‐by‐side advice at the time of the tests, as many of the participants were not familiar with a touch screen and stylus. CogEvo calculates scores automatically based on the speed and accuracy of the task performance.

### *Statistical analysis*

Descriptive statistics (e.g. mean, standard deviation, prevalence rates) were generated initially for the participants’ basic characteristics and CogEvo performance. CogEvo z‐scores were calculated from the total of five test values after the mean and standard deviation were calculated using the test results of the CN group. Analysis of variance (anova) was used to compare the age and years of education among the CN, MCI and mild AD groups. Analysis of covariance (ancova) was used to compare MMSE and CogEvo z‐scores among the three groups after adjusting for age and years of education. To verify the reliability of CogEvo, 30 participants (12 CN, 8 MCI and 10 mild AD patients) were retested at intervals of approximately 2 months, and intraclass correlation coefficient tests were carried out. To verify the validity of CogEvo, we examined the Pearson correlation coefficients between CogEvo and MMSE scores using data from the outpatients. To confirm its usefulness in screening for normal and mild cognitive decline (MCD; MCI plus mild AD), CogEvo was analyzed using logistic regression analysis after adjusting for age, sex and years of education. Receiver operating characteristic (ROC) analysis was also carried out. The areas under ROC curves (AUC) were used to compare the usefulness of tests. Finally, cut‐off values and sensitivity and specificity were calculated.

## Results

**Figure 1:**
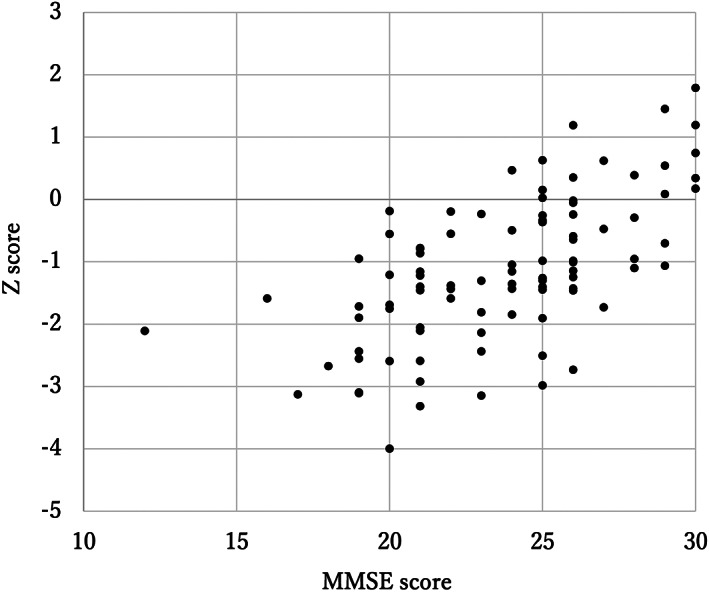
Correlation between Mini‐Mental State Examination (MMSE) and CogEvo z scores. The correlation between MMSE and CogEvo z scores in the Alzheimer's disease, mild cognitive impairment and cognitively normal groups is shown.

The study participants were 95 outpatients (40 mild AD, 38 MCI and 17 CN), and 71 individuals who accompanied these patients to the hospital and were judged as being CN, resulting in a total of 88 CN participants. Table 1 shows the participants’ basic characteristics and test results. Test–retest examinations were carried out on 30 patients, and the results were excellent (intraclass correlation coefficient 0.892, 95% confidence interval 0.773–0.949; *P* < 0.001). When a correlation was observed between MMSE and CogEvo z‐scores for AD, MCI and CN in the patient groups, the correlation coefficient was 0.616 (*P* < 0.001), indicating the validity of CogEvo as a cognitive function assessment test (Fig. 1).

**Table 1:** Characteristics of the participants

	AD (*n* = 40)		MCI (*n* = 38)		CN (*n* = 88)		Total (*n* = 166)		*P*
	Mean	SD	Mean	SD	Mean	SD	Mean	SD	
Age (years)	81.3^‡^	4.1	78.6^‡^	8.0	74.2	8.1	76.9	7.9	<0.001
Sex, female (%)^§^	60.0		60.5		65.9		63.3		0.752
Education (years)	10.9^‡^	2.7	12.1	3.0	12.6	2.8	12.0	2.9	0.007
MMSE^¶^	20.4^‡†^	2.2	25.5^‡^	1.6	27.6	2.0	23.7	3.5	<0.001
CogEvo z‐score	−1.9^‡†^	0.9	−0.8^‡^	0.8	0.0	1.0	−0.6	1.2	<0.001

The results of post‐hoc Bonferroni analysis are as follows: ^**‡**^ *P* < 0.05 compared with cognitively normal (CN), ^**†**^ *P* < 0.05 compared with mild cognitive impairment (MCI), ^**§**^χ^2^ analysis was applied. ^**¶**^As for the Mini‐Mental State Examination (MMSE) scores for the CN and Total groups, the data from outpatients (CN = 17, Total = 95) were calculated and analyzed. anova was used to compare the age and years of education, and ancova was used to compare MMSE and CogEvo z‐score adjusting age and years of education among the CN, MCI and mild Alzheimer's disease (AD) groups, respectively.

**Figure 2:**
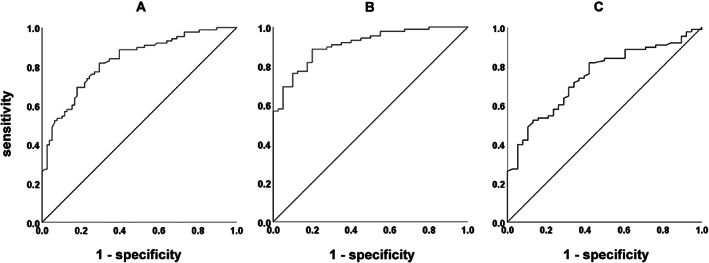
(a) Area under the receiver operating characteristic curve analysis differentiating mild cognitive decline from cognitively normal people based on CogEvo scores. Receiver operating characteristic curve analysis differentiating the (b) mild Alzheimer's disease and cognitively normal groups, and the (c) mild cognitive impairment and cognitively normal groups are also shown.

Logistic regression analysis was carried out after adjusting for age, sex and years of education as dependent variables in the MCD (*n* = 78) and CN groups (*n* = 88). CogEvo scores were shown to be effective to differentiate between these two groups (*P* < 0.001; Table 2). The results of the ROC analysis showed moderate accuracy (AUC 0.830) in differentiating the MCD from the CN group based on CogEvo scores (Fig. 2a). The AUCs of the ROC analyses of the comparison of the two groups of five subtests were as follows: AUC 0.833 for “Orientation”, 0.697 for “Visual search”, 0.719 for “Flash light”, 0.724 for “Route 99” and 0.726 for “Just fit”. When the mild AD (*n* = 40) and CN groups (*n* = 88) were analyzed, the AUC was 0.909, indicating high accuracy (Fig. 2b). When the MCI (*n* = 38) and CN groups (*n* = 88) were analyzed, the AUC was 0.747, indicating moderate accuracy (Fig. 2c). The sensitivity and specificity at optimal cut‐off values were 81.8% and 70.5% at z = −0.946 in the MCD and CN groups, respectively, and 88.6% and 80.0% at z = −1.156 in the mild AD and CN groups, respectively. The sensitivity and specificity at optimal cut‐off values for the MCI and CN groups were 81.8% and 57.9%, respectively, at z = −0.948. ROC analysis of CogEvo and MMSE scores applied only to the MCD outpatients (AD *n* = 40, MCI *n* = 38), and the CN outpatients group (*n* = 17) showed moderate accuracy (CogEvo 0.826 *vs* MMSE 0.899, respectively).

**Table 2:** Logistic regression analysis showing the usefulness of CogEvo in differentiating mild cognitive dysfunction from cognitively normal

	OR	95% CI		*P*
Age	0.974	0.912	1.039	0.424
Sex	0.925	0.403	2.123	0.855
Education	1.031	0.896	1.187	0.668
CogEvo z‐score	3.59	2.201	5.855	<0.001

CN, cognitively normal; CI, confidence interval; MCD, mild cognitive decline; OR, odds ratio.

## Discussion

CogEvo was shown to have reliability and validity as a computer‐based cognitive function test, with significant differences in scores between the mild AD, MCI and CN groups, thereby verifying its usefulness for the identification of early cognitive decline. ROC analysis of the discrimination between the MCD group with mild AD and MCI and the CN group showed sufficient discriminating power. CogEvo is therefore expected to be useful for discriminating the presence or absence of cognitive decline.

Numerous attempts have been made to develop cognitive function tests using computers.^6^, ^7^, ^8^, ^21^, ^22^ Such tests are assumed to have several strong points. First, they can be carried out without an examiner by providing automatic instructions on the screen or computer voice commands to reduce the burden of the examiner. Second, variation due to different examiners can be reduced. Third, data can be stored digitally and simultaneously in the computer during testing. Fourth, the reaction time, a parameter for assessing performance, can be easily measured and recorded by the computer. Fifth, as there is a possibility that digital‐based tests can be carried out via the Internet, the tests can be carried out at home. In addition, CogEvo has the advantage of computer‐based cognitive function testing, which can create a variety of similar patterns in each test. Therefore, people can enjoy it similar to a game every day. Furthermore, among the computerized cognitive assessment tools, the possibility for the use of cognitive training is the most advantageous point of CogEvo, and it becomes quite popular and widely used as a cognitive training tool in Japan, although the efficacy has not been fully studied.

In contrast, there are several disadvantages associated with using a computer. First, such tests might be difficult for people with poor vision, especially older people. Second, many older people are unfamiliar with computer operation. Third, although CogEvo has the advantage that it can create similar patterns repeatedly, it is possible that when doing so, differences in difficulty can arise. In the present study, however, it was considered that a stable pattern in regard to the difficulty of tests was achieved, because sufficient correlation was observed in the test–retest examinations.

In the present study, five of 12 CogEvo tests were selected related to five cognitive domains. Although recent memory tasks have generally been recognized as being the most useful for the differential diagnosis of early dementia, these were not included in the repertoire of CogEvo. However, among the five tasks used in the present CogEvo set, the time orientation task was included. In general, temporal orientation is known to decrease in the early stages of dementia, as well as or more than that in recent memory tasks.^23^, ^24^, ^25^ In fact, the CogEvo orientation task had the best value among the subtests in the ROC analysis (data not shown). The use of this task in CogEvo can serve as a substitute for recent memory tasks, and might contribute to a differential diagnosis of early‐stage dementia from CN older people.

In the present study, we did not verify the application of CogEvo as a cognitive training instrument. However, many of the participants enjoyed it similar to a game, even during testing. It was also observed that different patterns in each test were created stably each time. Therefore, it was considered that CogEvo could be applied to daily cognitive training and periodic cognitive function checks. Although few satisfactory reports have been published on the feasibility of continuing computer training, it has been reported that, as a factor on the part of the user, having experience with computers is associated with continuation.^12^

Although factors on the user side are important, those on the software side, such as the content of the cognitive training and the ease of use, also need to be examined. In the future, it is expected that a comparison of the continuation rates among computer‐based cognitive training programs will be carried out. In addition, it will be necessary to explore the target population for training, the duration of training and the measurement methods to assess training outcomes.

The present study had several limitations. First, the cognitive domain of the assessment could be reconsidered. The most important factor in differentiating early dementia is considered to be a recent memory test, but it is technically difficult to carry out a delayed memory task as a computer‐based test. In the future, it will be necessary to consider adding a delayed recognition task as a substitute for the delayed memory test in CogEvo. In addition, a delayed memory test using voice inputs for answering should be considered. Second, although CogEvo is expected to be useful in daily cognitive training, verification is required in conjunction with the use of CogEvo as an assessment tool. Third, in the present study, we examined three groups: CN, MCI due to AD and mild AD. It will be necessary to examine whether CogEvo can be applied to other types of cognitive impairments, such as vascular or Lewy body dementia. Fourth, in the present study, data from 88 normal participants were used for standardization. More extensive tests that serve to standardize CogEvo by age and years of education need to be carried out.

In conclusion, here, we reported the usefulness of CogEvo, a computer‐based cognitive assessment tool, designed for both cognitive assessment and training. With the rapid increase in the number of patients with dementia and aging populations around the world, the development and daily use of such tools can be expected to advance further.

## Disclosure statement

The authors declare no conflict of interest.
